# *Aspergillus fumigatus* strains that evolve resistance to the agrochemical fungicide ipflufenoquin in vitro are also resistant to olorofim

**DOI:** 10.1038/s41564-023-01542-4

**Published:** 2023-12-27

**Authors:** Norman van Rhijn, Isabelle S. R. Storer, Mike Birch, Jason D. Oliver, Michael J. Bottery, Michael J. Bromley

**Affiliations:** 1 Manchester Fungal Infection Group, Division of Evolution, Infection, and Genomics, Faculty of Biology, Medicine and Health, The University of Manchester, Manchester, UK; 2 F2G Ltd, Manchester, UK

**Keywords:** Fungal evolution, Fungal biology

## Abstract

Widespread use of azole antifungals in agriculture has been linked to resistance in the pathogenic fungus *Aspergillus fumigatus*. We show that exposure of *A. fumigatus* to the agrochemical fungicide, ipflufenoquin, in vitro can select for strains that are resistant to olorofim, a first-in-class clinical antifungal with the same mechanism of action. Resistance is caused by non-synonymous mutations within the target of ipflufenoquin/olorofim activity, dihydroorotate dehydrogenase (DHODH), and these variants have no overt growth defects.

## Abstract (web summary)

The agrochemical fungicide ipflufenoquin can drive cross-resistance to the clinical antifungal olorofim in *Aspergillus fumigatus* in vitro.

## Main

Invasive aspergillosis (IA), a disease caused primarily by the saprotrophic fungus *Aspergillus fumigatus*, is estimated to cause 500,000 deaths each year (http://www.gaffi.org/why/burden-of-disease-maps/). The triazole class of antifungals represents first-line treatments for IA and effectively cures disease in the majority of patients^1,2^. Correlative data indicate a link between the use of analogous compounds for crop protection and the emergence of triazole resistance in *A. fumigatus*^3–6^. In some centres, resistance rates now exceed 20% and 9 in 10 patients infected with a resistant strain will succumb to the infection if alternate therapies are not given rapidly^7–10^. Olorofim, a next-generation antifungal in the orotomide class, is currently in phase III clinical trials for IA and other mould infections and is active against azole-resistant isolates^11^. Orotomides act by disrupting pyrimidine synthesis via inhibition of dihydroorotate dehydrogenase (DHODH)^11,12^. As olorofim nears clinical deployment, ipflufenoquin, a fungicide that has been shown to be a potent inhibitor of DHODH activity in *Neurospora crassa*^13^, has been approved by the US Environmental Protection Agency for use in agriculture^14^ and is under review in other territories^15^. There is clear concern that the widespread use of ipflufenoquin could drive resistance to the orotomides in *A. fumigatus*.

**Fig. 1:**
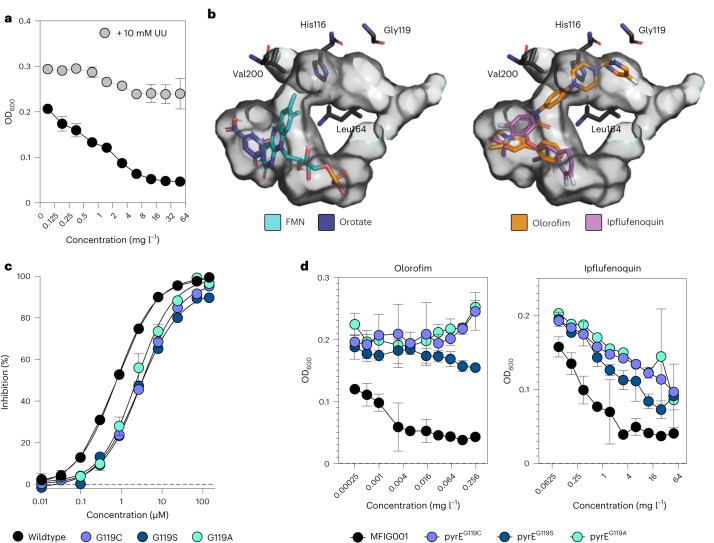
Ipflufenoquin acts against DHODH. **a**, MIC determination of ipflufenoquin (0.175–50 mg l^−1^) against *A. fumigatus* MFIG001 in RPMI-1640 (black) using the EUCAST methodology and with addition of 10 mM uridine and uracil (grey). OD_600_ was measured after 48 h. Three biological replicates were assessed. Data are presented as mean ± s.e.m. **b**, Molecular docking of FMN and orotate (left in cyan and blue, respectively) and olorofim and ipflufenoquin (right, orange and purple, respectively) to an Alphafold2 model of *A. fumigatus* DHODH. The pocket of the active site is shown in grey. **c**, Protein inhibition assay of ipflufenoquin towards wildtype and G119 variants of DHODH. At least five biological replicates were assessed. Data are presented as mean ± s.e.m. (*n* = 5). **d**, MIC determination of G119 variants (G119C, G119S and G119A) against ipflufenoquin (right) and olorofim (left) according to EUCAST methodology. OD600 was measured after 48 h. Three biological replicates were assessed. Data are presented as mean ± s.e.m. Source data

**Extended Data Fig. 1:**
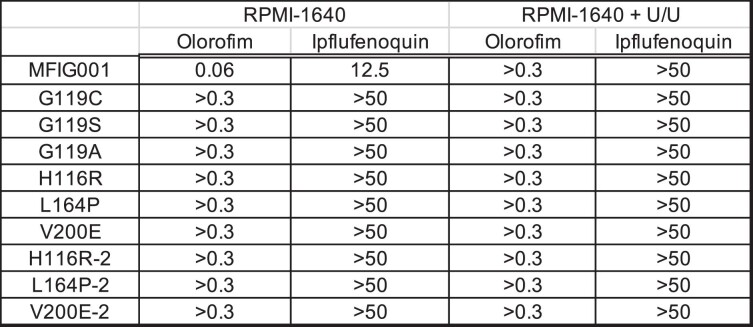
MIC determination of olorofim and ipflufenoquin. MIC determination of olorofim and ipflufenoquin against G119 variants of *A. fumigatus*. Three biological replicates were assessed.

**Extended Data Fig. 2:**
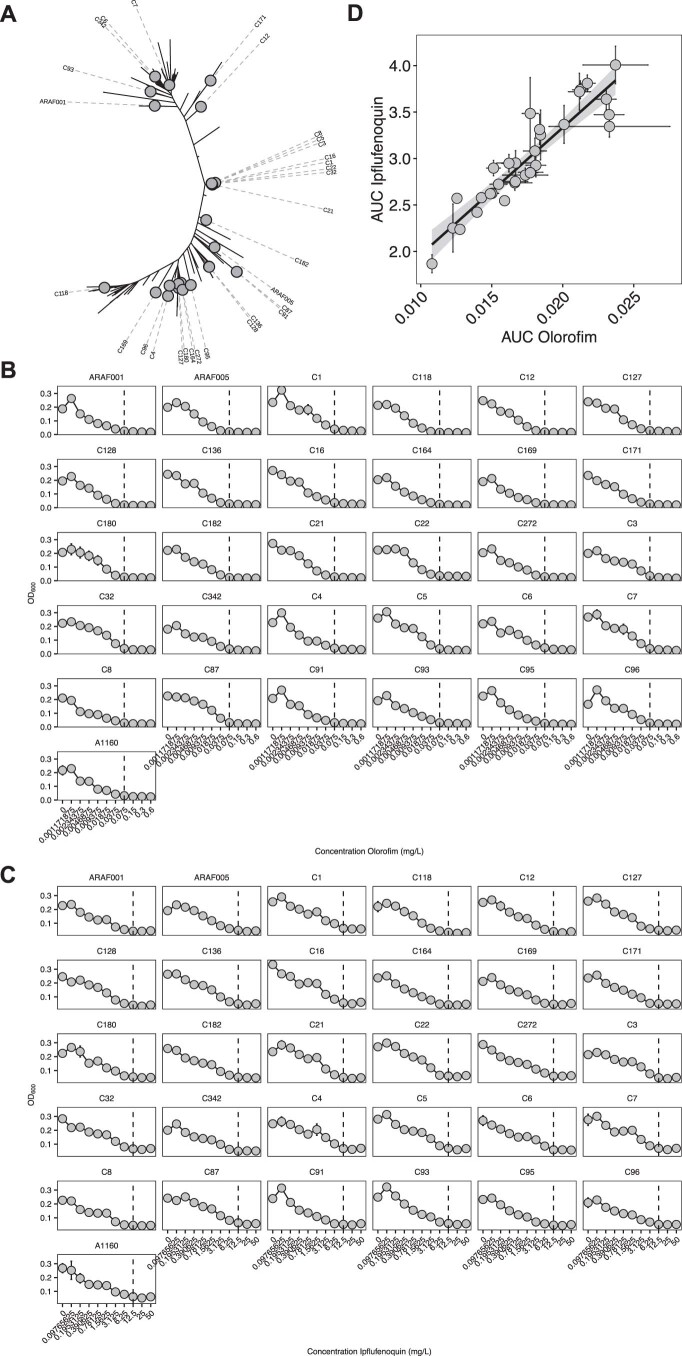
MIC determination for natural *A. fumigatus* isolates of olorofim and ipflufenoquin. A: Phylogenetic tree highlighting natural *A. fumigatus* isolates used (n = 30) sampled across a wide genetic diversity of the species, tree generated in Rhodes et al.^5^. B and C: MIC determination of olorofim (B) and ipflufenoquin (C) against thirty natural *A. fumigatus* isolates. Three biological replicates were assessed per isolate. Data are presented as the mean with SEM. The MIC is shown as a dotted line. D: Correlation of the AUC to ipflufenoquin and olorofim of the 30 natural *A. fumigatus* isolates. Three biological replicates were assessed per isolate. Data are presented as the mean with SEM. Source data

Our growth inhibition studies confirm that ipflufenoquin is active against *A. fumigatus* (minimum inhibitory concentration (MIC) 12.5 mg l^−1^) at levels that are below the concentration of its use in crop protection (50 mg l^−1^)^16^, and consistent with ipflufenoquin acting exclusively by inhibiting DHODH, drug activity can be completely reversed by the addition of exogenous pyrimidines (Fig. 1a and Extended Data Fig. 1). The activity of ipflufenoquin was consistent across a collection of 30 natural isolates (Extended Data Fig. 2a) (12.5 mg l^−1^) and similarly, all isolates had the same MIC to olorofim of 0.075 mg l^−1^ (Extended Data Fig. 2b,c). By comparing the area under the curve (AUC) calculated from the drug dose-response curves, we were able to detect a correlation in the ability of strains to grow in sub-MICs of olorofim and ipflufenoquin (Extended Data Fig. 2d; *R*^2^ = 0.7731, *P* = 7.8 × 10^−12^).

**Extended Data Fig. 3:**
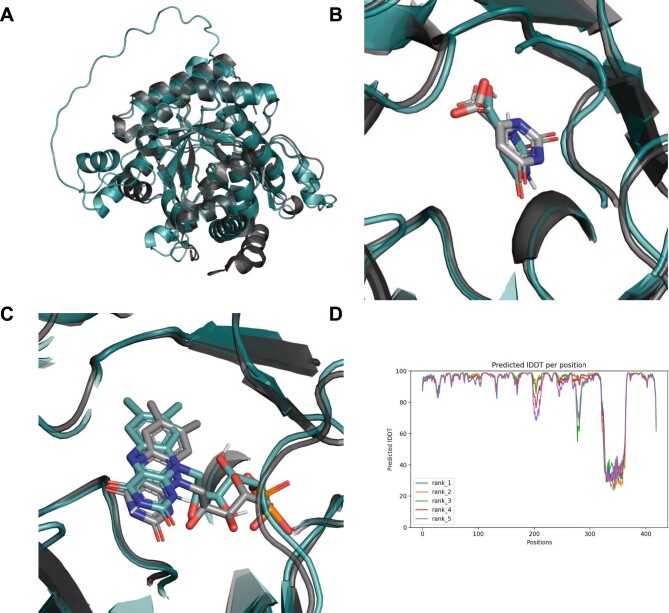
An Alphafold2 model of *A. fumigatus* DHODH. A: superposition of human DHODH (PDB: 2PRH_1, grey) and *A. fumigatus* DHODH (teal). The *Af*DHODH modelled region was Asp110-Leu523, which included a region of low per-residue confidence scores (pIDDT), Arg426-Val478, which is shown as a long loop. hDHODH and *Af*DHODH share 35% identify and 84% coverage across the full-length sequences. B: co-crystal structure of hDHODH (grey) in complex with orotate, shown in grey. The predicted pose of orotate from molecular docking with *Af*DHODH (teal) is superimposed. C: co-crystal structure of hDHODH (grey) in complex with FMN, shown in grey. The predicted pose of FMN from molecular docking with *Af*DHODH (teal) is superimposed. D: Predicted plDDT for 5 predicted structures from AlphaFold2. We used the rank 1 model. A score of <50 is considered very low confidence. Models show a region of low per-residue confidence, Arg316-Val368, which is shown as a long loop (A). However, this region was distal to the binding pocket discussed and therefore not of functional relevance to this work.

**Extended Data Fig. 4:**
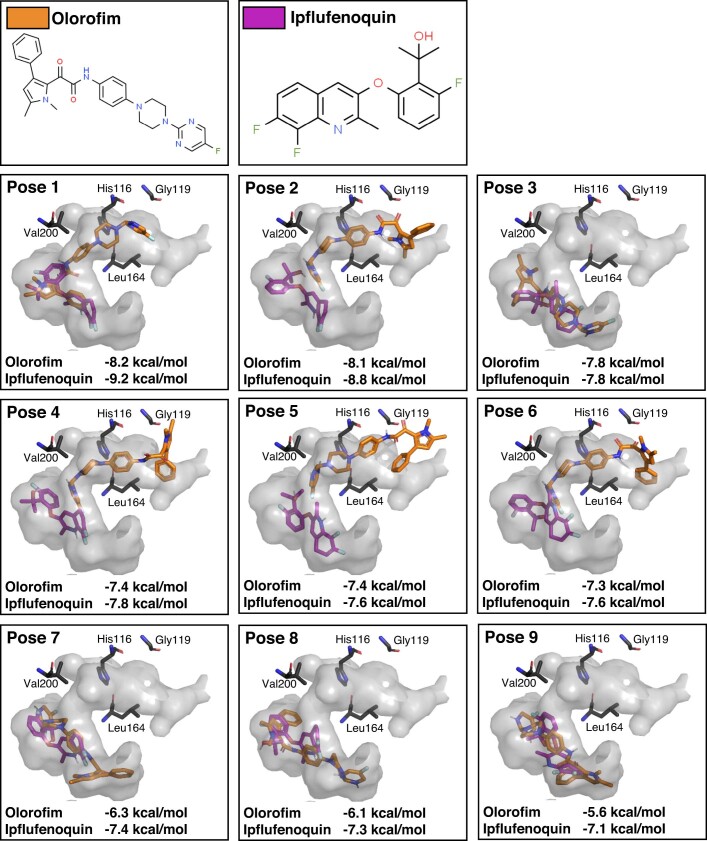
Rank 9 AfDHODH docking models. Olorofim and ipflufenoquin were docked to the predicted active site of *Af*DHODH using VSPipe with the standard parameters. The top 9 poses are shown with olorofim in magenta and ipflufenoquin in orange. Binding energies and residues relevant for decreased susceptibility to olorofim and ipflufenoquin are displayed for each pose.

Using an Alphafold2 generated model of the *A. fumigatus* (*Af*)DHODH (Extended Data Fig. 3), we predict that both ipflufenoquin and olorofim bind effectively within the active site of the enzyme (binding energies: orotate -7.0 kcal mol^−1^, flavin mononucleotide (FMN) −10.8 kcal mol^−1^, olorofim −8.2 kcal mol^−1^, ipflufenoquin −9.2 kcal mol^−1^) (Fig. 1b). The most energy-favourable poses indicate that the larger olorofim molecule but not ipflufenoquin extends from the active site across the entry channel (Extended Data Fig. 4). Consistent with the model, ipflufenoquin directly inhibits the enzymatic activity of *Af*DHODH, although the half maximal inhibitory concentration (IC_50_)is more than 15 times higher than that previously observed for olorofim (IC_50_: 0.77 µM ipflufenoquin vs 0.044 µM olorofim; Fig. 1c)^11,17^.

**Extended Data Fig. 5:**
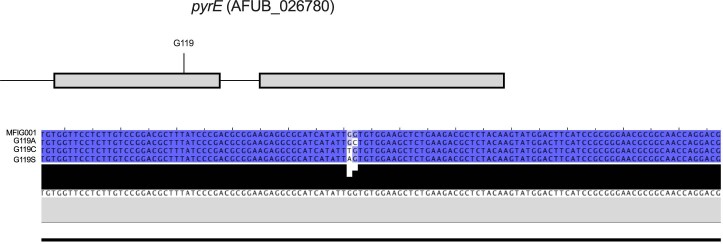
Sanger sequencing validation of G119 variants. The *pyrE* gene was amplified by PCR and Sanger sequencing over the G119 position was performed. No other mutations within the *pyrE* coding region were observed. Source data

Non-synonymous small nucleotide polymorphisms (SNPs) at bases 355 (G to A/T) and 356 (G to T) of the *pyrE* gene equivalent to amino acid position G119 (G119C, G119S or G119A) at the entrance to the active site (Fig. 1b) of (*Af)*DHODH are sufficient to provide high-level resistance (>8 mg l^−1^) to olorofim^17^. To assess whether these mutations result in cross-resistance to ipflufenoquin, we generated isolates containing the variants (Extended Data Fig. 5)^18^. All variants were resistant to the highest concentration of olorofim tested in our assay (>0.3 mg l^−1^) (Fig. 1d and Extended Data Fig. 1). Although our predictions indicate that ipflufenoquin does not interact directly with G119, these mutants were cross-resistant to ipflufenoquin (>50 mg l^−1^) (Fig. 1d and Extended Data Fig. 1), suggesting that G119 mutations either result in a conformational change of the active site that affects the ability of ipflufenoquin to bind or restricts access to the active site via the entry channel. Mirroring this, *Af*DHODH protein variants G119C, G119S and G119A had marked increases in IC_50s_ to olorofim (IC_50_ >100 µM^17^) and ipflufenoquin (IC_50_ 3.3 µM, 3.3 µM and 2.5 µM, respectively; Fig. 1c).

**Extended Data Fig. 6:**
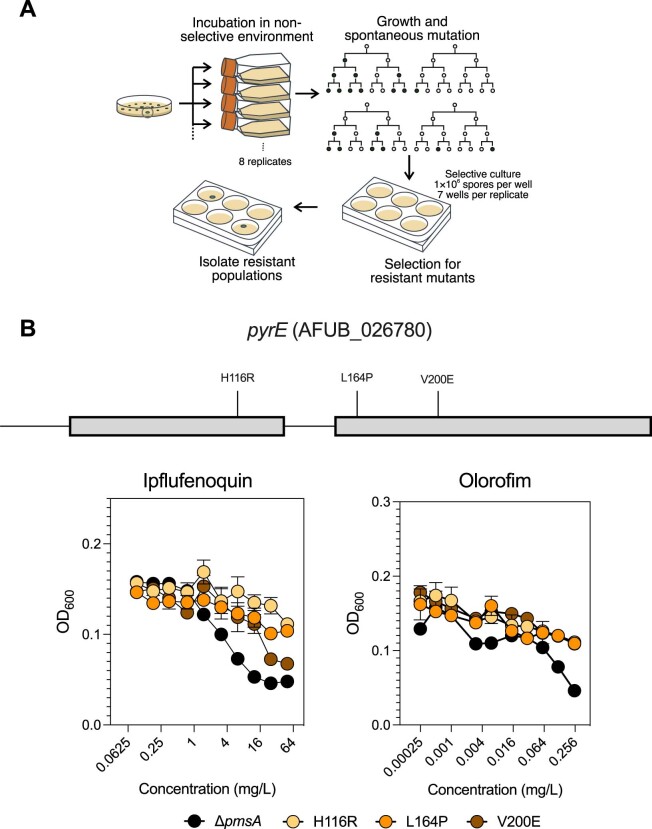
Determination of ipflufenoquin and olorofim against *pmsA* mutants. A) workflow of fluctuation assays in *A. fumigatus*. Non-selective environment generate a genetically diverse population, for which resistant mutants are selected for on ipflufenoquin containing medium. B) MIC determination of olorofim and ipflufenoquin against G119 variants of *A. fumigatus* according to EUCAST methodology. OD_600_ was measured after 48 hours. Three biological replicates were assessed. Data are presented as the mean with SEM.

**Extended Data Fig. 7:**
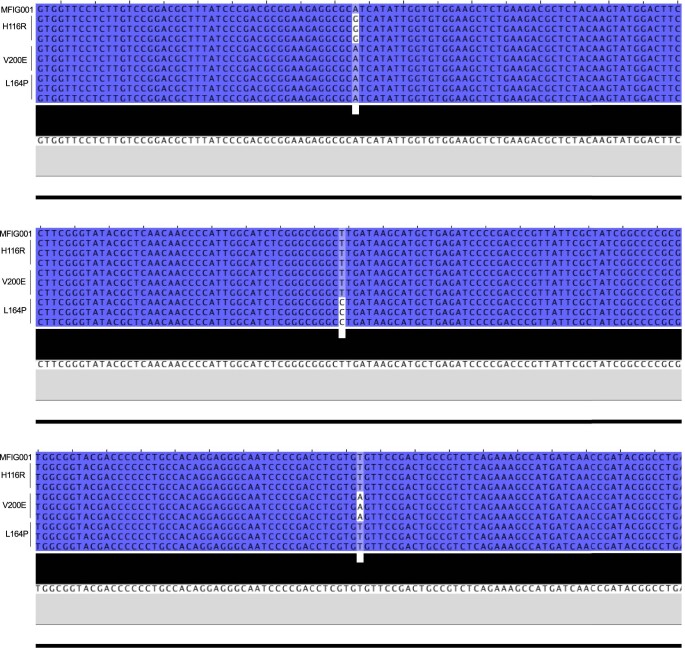
Sanger sequencing validation of H116R, L164P and V200E variants. The *pyrE* gene was amplified by PCR and Sanger sequencing over the H116, L164 and V200 position was performed. No other mutations within the *pyrE* coding region were observed. Source data

Using a hypermutator strain of *A. fumigatus* (∆*pmsA* (*AFUB_029050*); orthologue of *E. coli* mutL), we identified 9 ipflufenoquin-resistant isolates (Extended Data Fig. 6a). Sequencing of the *pyrE* gene revealed SNPs at three loci: H116R (*n* = 3 isolates), L164P (*n* = 3) and V200E (*n* = 3) (Extended Data Fig. 7). These mutants were resistant to ipflufenoquin (>50 mg l^−1^) and olorofim at levels exceeding the published MIC_90_ for *A. fumigatus* (>0.3 mg l^−1^) (Extended Data Figs. 1 and 6b)^19^.

**Fig. 2:**
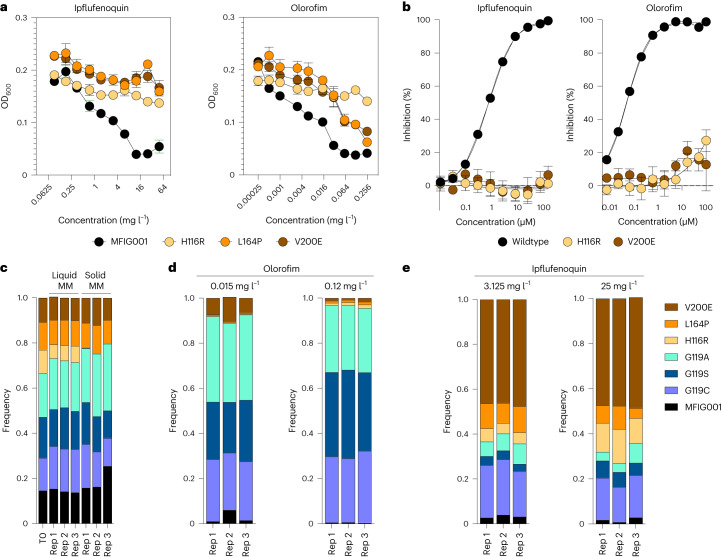
Ipflufenoquin can select for olorofim-resistant *A. fumigatus*. **a**, MIC determination of H116R, L164P and V200E variant mutants against ipflufenoquin and olorofim according to EUCAST methodology. OD_600_ was measured after 48 h. Three biological replicates were assessed. Data are presented as mean ± s.e.m. **b**, Protein inhibition assay of ipflufenoquin towards wildtype, V200E and H116R variants of DHODH. At least five biological replicates were assessed. Data are presented as mean ± s.e.m. (*n* = 5). **c**, Frequency of mutants after mixed-inoculum experiments to determine basal fitness of each mutant on solid and liquid MM medium. Three biological replicates were assessed and individual data from replicates are shown. **d**, Frequency of mutants after low (0.015 mg l^−1^) and high (0.12 mg l^−1^) olorofim challenge in a mixed-inoculum competition assay. Three biological replicates were assessed and individual data from replicates are shown. **e**, Frequency of mutants after low (3.125 mg l^−1^) and high (25 mg l^−1^) ipflufenoquin challenge in a mixed-inoculum competition assay. Three biological replicates were assessed and individual data from replicates are shown. Source data

**Extended Data Fig. 8:**
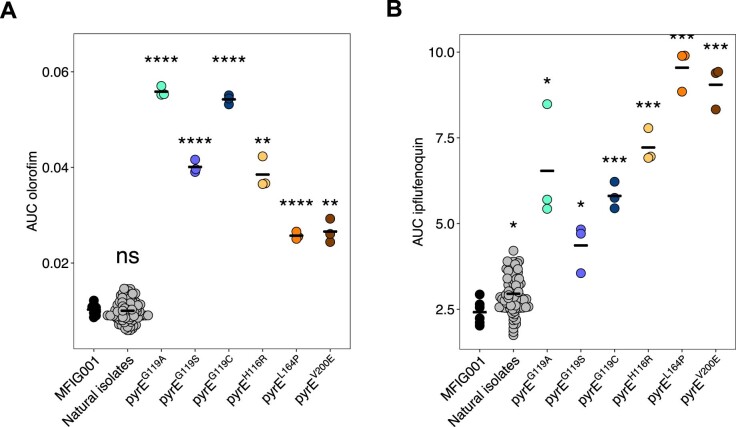
AUC analysis of *A. fumigatus* isolates to olorofim and ipflufenoquin. AUC of MIC curves to A: olorofim and B: ipflufenoquin presented in Figs. 1, 2 and Extended Data Fig. 2. Each point represents an independent replicate (N = 3 for *pyrE* mutants, N = 90 for natural isolates, N = 6 for MFIG001). Crossbar shows the mean within groups. Significant differences between groups calculated using two-sided *t*-tests with Bonferri correction for multiple comparisons, * < 0.05, ** < 0.01, *** < 0.001, (ANOVA A: olorofim F_7,109_ = 511, p < 0.0001, B: ipflufenoquin F_7,109_ = 141, p < 0.0001 (pairwise comparisons in Source Data).

To confirm that the polymorphisms identified in our hypermutator isolate were directly linked to the ipflufenoquin-resistance phenotype, we reconstructed the SNPs in an isotype background (*A. fumigatus* MFIG001)^17^. Strains harbouring the three variants (*n* = 2 independent isolates for each) displayed high-level resistance to ipflufenoquin (MIC > 50 mg l^−1^) (Fig. 2a and Extended Data Fig. 1), were cross-resistant to olorofim at the highest concentrations tested (MIC > 0.3 mg l^−1^) (Fig. 2a and Extended Data Fig. 1) and exceeded the MIC observed for any of the natural isolates tested. Evaluation of the AUC from the drug dose-response curves revealed significant decreases in the susceptibility of the mutants to the inhibitors at sub-MIC levels when compared with the isotype strain (Extended Data Fig. 8) and the natural isolate collection. The mutants isolated from olorofim (G119A and G119C) were less susceptible to olorofim than those selected on ipflufenoquin (H116R, L164P and V200E) and vice versa (Extended Data Fig. 8 and Supplementary Table 1). Enzyme activity of *Af*DHODH proteins with the variants H116R and V200E was only modestly inhibited by either ipflufenoquin or olorofim even at the highest concentration tested (IC_50_ >100 µM; Fig. 2b).

**Extended Data Fig. 9:**
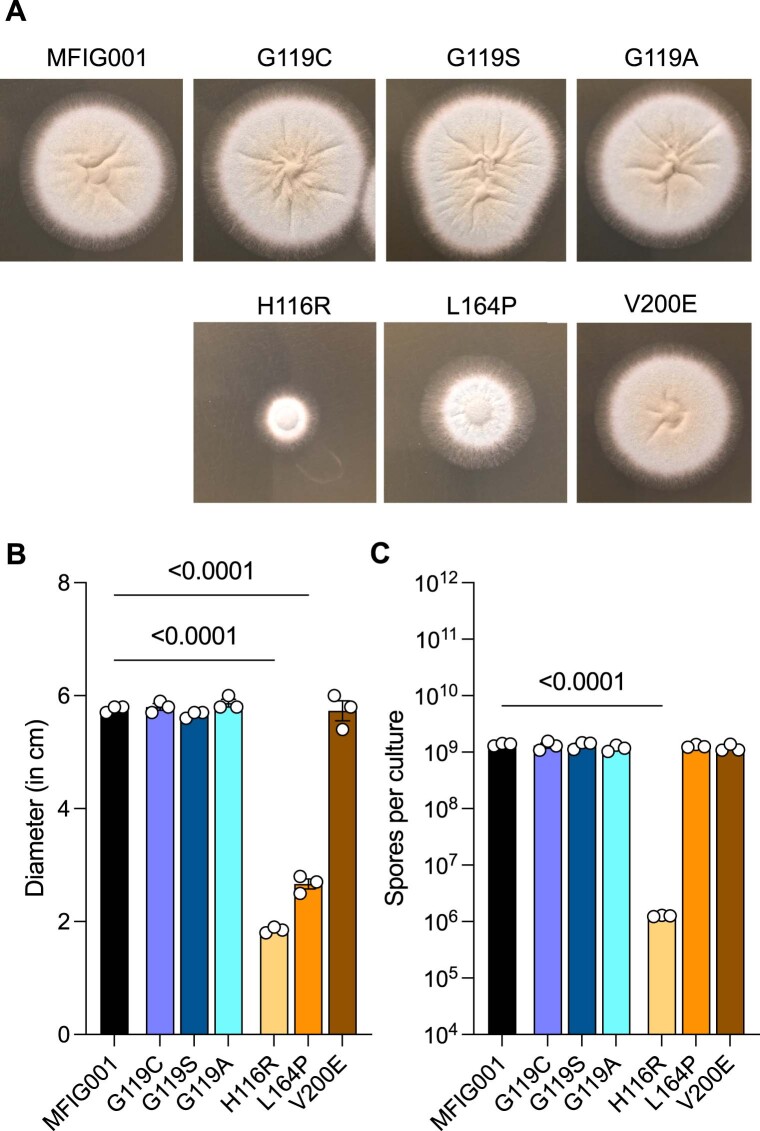
Phenotypic characterisation of resistant mutants. A) Representative images of spot assays on Sabouraud agar medium of MFIG001 and mutant isolates. B) Radial growth was measured after 48 hours on Sabouraud agar medium. Data are presented as the mean ± s.d. of three experiments. *P* values were determined by two-sided ANOVA with Tukey multiple comparison between means from a sample size of *n* = 3. C) Sporulation was measured after 3 days by inoculating MM cultures with equal spores. Spores were harvested through Miracloth and counted on a haemocytometer. Data are presented as the mean ± s.d. of three experiments. *P* values were determined by two-sided ANOVA from a sample size of *n* = 3. Source data

**Extended Data Fig. 10:**
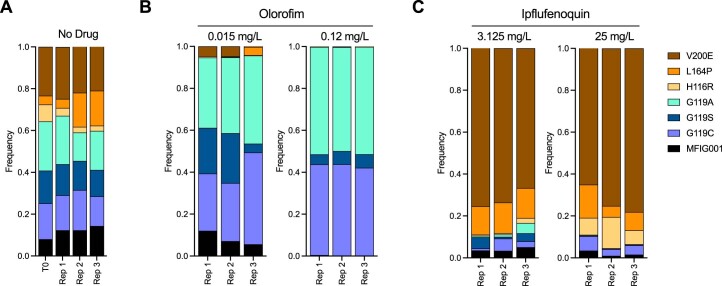
Competitive fitness in liquid RPMI-1640. A) Relative abundance of MFIG001 and mutants in RPMI-1640. Abundance was calculated as the amount of reads per isolate normalised to total QC-passed sequencing reads. Data are presented for independent replicates of three experiments. B) Abundance of MFIG001 and mutants in low (0.015 mg/L) and high (0.12 mg/L) olorofim challenge in a mixed inoculum competition assay. Data are presented for independent replicates of three experiments. C) Abundance of MFIG001 and mutants in low (3.125 mg/L) and high (25 mg/L) ipflufenoquin challenge in a mixed inoculum competition assay. Data are presented for independent replicates of three experiments.

Mutations that confer resistance are often associated with a cost to fitness or reduced sporulation that impact the ability of strains to become widely distributed. Radial growth assays of the resistant mutants showed that only strains with the H116R and L164P variants had reduced growth rates (Extended Data Fig. 9a,b; *P* < 0.0001 for both variants), while only the mutant harbouring the H116R variant had a significant sporulation defect (*P* < 0.001) (Extended Data Fig. 9c). To model the fitness of the resistant variants in a competitive context, we created a mixed inoculum with isolates that harbour the 6 DHODH variants and the parental MFIG001 isolate, allowed the strains to compete in co-culture, and using a process analogous to Bar-seq^20^, we evaluated the abundance of each strain at the beginning and end of the experiment. In the absence of drug pressure, the H116R variant showed reduced competitive fitness in both liquid and solid Aspergillus minimal media (AMM), replicating the outcome of the radial growth and sporulation experiments (Fig. 2c); however, no competitive disadvantage was observed for the other strains. Upon exposure to sub-inhibitory concentrations of olorofim (0.015 mg l^−1^) in solid media, the G119C, G119A and G119S variants increased in frequency while the H116R, L164P and V200E variants were detected at a lower frequency, suggesting that the latter variants are less fit than the G119 variants under olorofim selection (Fig. 2d). At higher concentrations of olorofim (0.12 mg l^−1^), the selective advantage of the G119 variants was further exacerbated (Fig. 2d; *P* < 0.001 by one-way analysis of variance (ANOVA)); however, the L164P, H116R and V200E had improved fitness when compared directly with the isotype strain (*P* = 0.038, *P* = 0.0314, *P* = 0.0312 by one-way ANOVA, respectively). Conversely, the isolate containing the V200E variant dominated populations that were exposed to ipflufenoquin (Fig. 2e). Notably, even under high ipflufenoquin stress, the G119C mutant had enhanced fitness when compared with the isotype control (*P* = 0.01). The H116R-containing strain also had enhanced fitness in ipflufenoquin in a dose-responsive manner despite its high inherent fitness cost (Fig. 2e). The ability of the G119 mutants to dominate the population under olorofim exposure and the V200E isolate under ipflufenoquin exposure was maintained in RPMI-1640 liquid culture (Extended Data Fig. 10). The L164P mutant, which exhibited a growth defect in isolated culture, did not appear to have a fitness defect when in competition, suggesting that nutrient exchange (cross-feeding) between strains may mitigate the growth impact caused by the mutation.

Our experiments are unable to replicate the complexities and vast variation that natural environmental settings pose; however, the lack of fitness defects in the laboratory settings tested here indicates that there are no obvious barriers to resistant strains surviving and becoming dominant in the environment. Furthermore, we cannot exclude the possibility that strains carrying variants that impact fitness, such as H116R, could accumulate compensatory mutations elsewhere in the genome that would ameliorate the initial fitness defect caused by the mutation.

It has been suggested that the widespread use of agricultural demethylase inhibitors is driving cross-resistance to the triazoles in *A. fumigatus*^3,5^. Our results show that the use of ipflufenoquin can similarly select for cross-resistance to the first-in-class orotomide, olorofim. The consequences of this are unclear; however, if resistance to the next-generation clinical antifungals is again driven by the use of agrochemicals, we will be limiting our future ability to treat *A. fumigatus* and potentially other filamentous fungal infections^11^. Despite the clear need for new fungicides to enhance food security^21^, we would advise that, before approval of ipflufenoquin for widespread commercial use, field trials are performed to (1) assess the likelihood that olorofim-resistant *A. fumigatus* will emerge via exposure to ipflufenoquin and (2) identify high-risk practices that have the potential to provide hotspots for resistance emergence and subsequent spread^22^.

## Methods

### Strains, culture conditions and antifungals

*A. fumigatus* MFIG001 was used as the parental isolate to generate all mutants by selection-free clustered regularly interspaced short palindromic repeats (CRISPR)-Cas9-mediated transformation^18,23^. *A. fumigatus* wildtype isolates (*n* = 30) representing the full genetic diversity within the population were provided by Matthew Fisher^5^. Unless otherwise stated, *A. fumigatus* strains used in this study were cultured on Sabouraud dextrose agar (Oxoid) for 3 d at 37 °C and collected in phosphate buffered saline (PBS) + 0.01% Tween-20. Olorofim was synthesized by Concept Life Sciences. Ipflufenoquin powder (>98% analytical grade) was obtained from CRM LabStandard. The fluctuation assay was performed by inoculating 1 × 10^6^ spores per well from 7 independent *A. fumigatus* ∆*pmsA* cultures into 8 wells containing *Aspergillus* MM medium^24^ + 5 mg l^−1^ ipflufenoquin. After 4 d of incubation at 37 °C, spores from wells containing growth were transferred to Sabouraud agar and grown at 37 °C for 3 d (Fig. 2a). Spores were collected in PBS + 0.01% Tween-20 and stored at −80 °C.

### Susceptibility testing

MIC determination for all drugs was carried out according to methods outlined by EUCAST (*n* = 3)^25^. Spores were inoculated per well of a CytoOne 96-well plate (StarLab) containing RPMI-1640 medium, 2.0% glucose and 165 mM MOPS buffer (pH 7.0) with a range of drug (olorofim 0.00025–0.3 mg l^−1^, ipflufenoquin 0.17–50 mg l^−1^). Plates were incubated at 37 °C for 48 h and optical density (OD) measurements were taken at 600 nm.

### Protein purification and activity assay

The cloning of *A. fumigatus* DHODH_(89–531)_ complementary DNA into protein expression vector pET44 (ref. ^11^) and the subsequent mutation of the Gly119 site to cysteine (G2S), valine (G2V), alanine (G2A) and serine (G2S) have been described previously^17^. His116 was mutated to arginine (H2R) and Val200 was mutated to glutamic acid (V2E) using the Phusion Site-Directed Mutagenesis kit (ThermoFisher) with pET44 *A. fumigatus* DHODH_(89–531)_ as the template. Primers were CGGAAGAGGCGCGTCATATTGGTGT and CGTCGGGATAAAGCGTCCGG for H2R and CCCGACCTCGTGAGTTCCGACTGCC and GATTGCCCTCCTGTGGCAGG for V2E mutagenesis. Expression of the various proteins in *E. coli* BL21 (DE3) cells (Merck) and purification by immobilized metal-affinity chromatography were followed by DHODH assay according to previously described protocol^11^. Mutagenesis of Lys164 to proline was also achieved, but the *E. coli* expressed protein was insoluble. DHODH assays were set up in the presence and absence of ipflufenoquin at concentrations between 0.01 and 144 µM. Assays were carried out in 50 mM Tris HCl (pH 8), 150 mM KCl, 10% (w/v) glycerol and 0.1% (w/v) Triton X-100 in the presence of 1 mM l-dihydroorotic acid, 0.05 mM coenzyme Q2 and 0.1 mM 2,6-dichloroindophenol as a redox indicator. Activity was determined by the decrease in absorbance at 600 nm and reaction velocities used to construct IC_50_ curves in XLfit 5.5.0 (IDBS).

### In silico protein prediction and docking

The structure of DHODH was determined using AlphaFold2 (ref. ^26^); for subsequent analysis, the highest-scoring model was used (pLDDT: 96.1, pTMscore: 0.9375). VSpipe^27^, a semi-automated pipeline that utilizes MGLTools (1.5.6) and AutoDock Vina (1.1.2), was used for targeted docking with the ligands and antifungals at the quinone binding pocket. The grid centre for the generated protein data bank (PDB) file was *x* = −2.582, *y* = −5.147 and *z* = −4.552. The grid spacing was set to the default of 0.375 Å and the box size was 30 × 30 × 30 Å^3^ to encompass the whole tunnel leading to the active site^28^. The 9 most-energy-favourable outputs (that is, with the lowest predicted binding free energy (ΔG)) were visualized in PyMOL 2.5 (Extended Data Fig. 4).

### Selection-free CRISPR-Cas9-mediated transformation

For transforming MFIG001, CRISPR RNAs (crRNAs) were designed to target the intron of the *pyrE* gene in A1163. The closest PAM site with scores >0.5, as calculated by EuPaGDT, was used, and 20-bp crRNAs were ordered from IDT (crRNA: GTATACCCGAAGACCTGCAT). CRISPR transformation was performed as previously described^18^. Briefly, *A. fumigatus* was grown overnight at 37 °C in Sabouraud agar, followed by protoplasting using Vinotaste. Protoplasts were washed twice in 0.6 M KCl, followed by resuspension in 0.6 KCl + 200 mM CaCl_2_. Guide RNA was formed by annealing crRNA to trans-activating CRISPR RNA (IDT) and ribonucleoproteins (RNPs) were formed by incubating at room temperature for 5 min with purified SpCas9 (IDT). The substrate for repair template synthesis (double-stranded (ds)DNA) was obtained by PCR amplification of *pyrE* from ipflufenoquin-resistant ∆*pmsA* isolates (primers: ACGCAAGAGGAACAGAGGAA and GGATGTTTCTGGGGAGGTTT). Single-stranded (ss)DNA repair template was obtained via lambda exonuclease degradation of the 5’ phosphorylated DNA strand. Any remaining dsDNA was degraded by Exonuclease I. This mixture was column purified (Geneflow). Single-strand repair template, RNPs and protoplasts were mixed with PEG-CaCl_2_ and incubated for 50 min on ice. PEG-CaCl_2_ (600 μl) was added, followed by incubation at room temperature for 20 min. Protoplasts were spread onto YPS medium, left at room temperature for 1 d, followed by incubation at 37 °C for 3 d. Transformants were purified and duplicate spot tests onto Sabouraud agar with and without antifungal (ipflufenoquin or olorofim) were performed to confirm resistance. Transformants were validated by Sanger sequencing the entire *pyrE* region introduced in the transformant isolates using primer AGTAAAGGAGGCACCCAAGAAAGCTGG (Genewiz).

### Phenotypic analysis

Spores (10^3^) of strains were spot inoculated onto the centre of solid Sabouraud agar plates. Plates were incubated for 48 h at 37 °C and measurements or images were taken. Sporulation assays were performed by culturing all isolates on Sabouraud dextrose agar for 4 d. Spore solutions were normalized to 5 × 10^6^ spores per ml, 50 µl was spread onto culture flasks containing AMM^24^ and incubated for 3 d at 37 °C. Spores were collected into 10 ml PBS + 0.01% Tween-20 through filtration over Miracloth and counted on a haemocytometer.

### Competition assays

Conidia were harvested from 3-day-old Sabouraud dextrose agar cultures using PBS + 0.01% Tween-20 (PBS-T) and collected by filtration through Miracloth (Millipore, 475855). Spores were quantified using a haemocytometer, normalized to 5 × 10^6^ spores per ml and pooled into one tube. For the solid AMM medium in vitro competitive fitness, the spore pool was diluted to 5 × 10^4^ spores per ml and 100 μl was plated onto solid AMM. Plates were incubated at 37 °C for 3 d. For the liquid RPMI-1640 in vitro competitive fitness assay, 1 ml of the pool of DHODH mutants and MFIG001 was added to each flask containing 10 ml RPMI-1640. All experiments were performed in triplicate and incubated in a shaking incubator at 120 r.p.m. for 24 h at 37 °C. For low and high olorofim concentrations, 0.015 mg l^−1^ and 0.12 mg l^−1^ were used, respectively, whereas for ipflufenoquin, 3.125 mg l^−1^ (low) and 25 mg l^−1^ (high) were used.

After incubation, plates and flasks were removed from the incubator and the spores (from each plate) or biomass (from each flask) were respectively collected using Miracloth and a Büchner funnel under vacuum. Liquid nitrogen was used to snap freeze the biomass, which was ground into a powder using a sterile mortar and pestle. From each baffled flask, 0.1 g was collected for DNA extraction. Total fungal DNA was extracted using a standardized CTAB DNA extraction^29^. Enrichment PCR (primers: TATGCTGTGGTTCCTCTTG and GTTGATCATGGCTTTCTGA) was performed using PhusionFlash polymerase and primers amplifying the *pyrE* gene for 1 cycle at 98 °C for 30 s, followed by 30 cycles at 98 °C for 10 s, 65 °C for 30 s, 72 °C for 30 s and a final extension step at 72 °C for 10 min. Enriched products were cleaned using AMPure beads and indexed with the Nextera XT kit (NEB) following manufacturer protocol. Sequencing was performed on an Illumina iSeq system following manufacturer protocol. Raw reads were quality controlled using FastQC and trimmed using Trimmomatic v.0.38.0. Trimmed reads were aligned to the A1163 *pyrE* gene using HISAT2 v.2.2.21; only reads over 145 bp were included to cover all SNPs. BAM files were exported to IGV v.2.8.13, from which the abundance of each SNP was assessed by counting the number of reads containing SNP-associated bases at individual genomic locations. A minimum of 8,000 reads per sample was analysed (range 8,281–135,184). The relative abundance of each strain was assessed using IGV by dividing the total reads containing an SNP by the total aligned reads for each sample.

### Statistical analysis

Statistical analysis was performed using Graphpad Prism 9 software. Differences among groups were analysed using a one-way ANOVA with Dunnett’s test. IC_50_ curves were analysed using XLfit 5.5.0 (IDBS).

### Reporting summary

Further information on research design is available in the Nature Portfolio Reporting Summary linked to this article.

### Supplementary information

Reporting Summary

Supplementary Table 1 Statistical analysis of AUC of olorofim and ipflufenoquin.

Supplementary Data 1 Alphafold2 model of DHODH.

### Source data

Source Data Fg. 1 Statistical source data.

Source Data Fig. 2 Statistical source data.

Source Data Extended Data Fig. 2 Statistical source data.

Source Data Extended Data Fig. 5 Statistical source data.

Source Data Extended Data Fig. 7 Statistical source data.

Source Data Extended Data Fig. 9 Statistical source data.

## Data Availability

Source Data are provided with this paper. Raw sequencing reads are available at SRA: PRJNA961782. Human DHODH PDB file was obtained from 2PRH_1 (https://www.rcsb.org/structure/2prh). Correspondence and requests for materials should be addressed to M. J. Bromley.
