# Supplementary material for: Aspergillus fumigatus strains that evolve resistance to the agrochemical fungicide ipflufenoquin in vitro are also resistant to olorofim
Source: Nat Microbiol. 2023 Dec 27;9(1):29–34. doi: 10.1038/s41564-023-01542-4 (PMC10769868; doi:10.1038/s41564-023-01542-4)
Supplement: Supplementary file 1 — Reporting Summary [file 41564_2023_1542_MOESM1_ESM.pdf]

nature portfolio

Corresponding author(s): Michael J. Bromley

Last updated by author(s): Oct 26, 2023

Reporting Summary

Nature Portfolio wishes to improve the reproducibility of the work that we publish. This form provides structure for consistency and transparency in reporting. For further information on Nature Portfolio policies, see our Editorial Policies and the Editorial Policy Checklist.

Statistics

For all statistical analyses, confirm that the following items are present in the figure legend, table legend, main text, or Methods section.

n/a	Confirmed	
☐	☒	The exact sample size (*n*) for each experimental group/condition, given as a discrete number and unit of measurement
☐	☒	A statement on whether measurements were taken from distinct samples or whether the same sample was measured repeatedly
☐	☒	The statistical test(s) used AND whether they are one- or two-sided<br>*Only common tests should be described solely by name; describe more complex techniques in the Methods section.*
☒	☐	A description of all covariates tested
☐	☒	A description of any assumptions or corrections, such as tests of normality and adjustment for multiple comparisons
☐	☒	A full description of the statistical parameters including central tendency (e.g. means) or other basic estimates (e.g. regression coefficient) AND variation (e.g. standard deviation) or associated estimates of uncertainty (e.g. confidence intervals)
☐	☒	For null hypothesis testing, the test statistic (e.g. *F*, *t*, *r*) with confidence intervals, effect sizes, degrees of freedom and *P* value noted<br>*Give P values as exact values whenever suitable.*
☒	☐	For Bayesian analysis, information on the choice of priors and Markov chain Monte Carlo settings
☒	☐	For hierarchical and complex designs, identification of the appropriate level for tests and full reporting of outcomes
☒	☐	Estimates of effect sizes (e.g. Cohen's *d*, Pearson's *r*), indicating how they were calculated

Our web collection on statistics for biologists contains articles on many of the points above.

Software and code

Policy information about availability of computer code

Data collection	The sequencing data were generated via the Illumina iSeq sequencing platform.
Data analysis	The sequencing data were trimmed using Trimmomatic version 0.38.0 and aligned to reference using HISAT2 version 2.2.1. Frequency of SNPs were analysed using IGV version 2.8.13. Statistical analysis was performed using Graphpad PRISM version 9. IC50 curves were analysed using XLfit 5.5.0 (IDBS). The structure of DHODH was determined using Alphafold 2 and docking was performed using VSpipe, which uses MGLTools v1.5.6 and AutoDock Vina v1.1.2. Protein structures were interpreted using PyMOL 2.5

For manuscripts utilizing custom algorithms or software that are central to the research but not yet described in published literature, software must be made available to editors and reviewers. We strongly encourage code deposition in a community repository (e.g. GitHub). See the Nature Portfolio guidelines for submitting code & software for further information.

Data

Policy information about availability of data

All manuscripts must include a data availability statement. This statement should provide the following information, where applicable:

- Accession codes, unique identifiers, or web links for publicly available datasets
- A description of any restrictions on data availability
- For clinical datasets or third party data, please ensure that the statement adheres to our policy

All source data is provided via supplementary files or a Source Data file for all Figures. Raw sequencing reads are available via SRA: PRJNA961782. Human DHODH PDB file was used from: 2PRH_1 (https://www.rcsb.org/structure/2prh)

Human research participants

Policy information about studies involving human research participants and Sex and Gender in Research.

Reporting on sex and gender	NA
Population characteristics	NA
Recruitment	NA
Ethics oversight	NA

Note that full information on the approval of the study protocol must also be provided in the manuscript.

Field-specific reporting

Please select the one below that is the best fit for your research. If you are not sure, read the appropriate sections before making your selection.

☒ Life sciences ☐ Behavioural & social sciences ☐ Ecological, evolutionary & environmental sciences

For a reference copy of the document with all sections, see nature.com/documents/nr-reporting-summary-flat.pdf

Life sciences study design

All studies must disclose on these points even when the disclosure is negative.

Sample size	No sample size calculations was required for this study but our sample sizes are similar to (or larger than) those reported in previous publications. Representative publications are mentioned below:<br><br>https://doi.org/10.1073/pnas.1608304113<br>https://doi.org/10.1038/s41467-019-14191-1
Data exclusions	No data were excluded from this study.
Replication	All experiments were conducted with independent biological replicates. The number of independent replicates were consistent with similar experiments conducted within the field. All experiments had a minimum of three independent biological replicates.Fluctuation tests were replicated 8 times in order to achieve 9 independent ipflufenequin resistant mutants.N values are reported in figure legends, and all plots show variation as SEM error bars
Randomization	No randomization was applied as this was impossible for the in vitro experimentation. Covariates were controlled by including relevant controls in experiments.
Blinding	Blinding and group allocations were not required or performed in this study. Data collection and analysis was performed by the same person who was not blinded to the conditions of the experiments. Biological experiments were impossible to conduct under the conditions of blinding. Hence, there was no blinding.

Reporting for specific materials, systems and methods

We require information from authors about some types of materials, experimental systems and methods used in many studies. Here, indicate whether each material, system or method listed is relevant to your study. If you are not sure if a list item applies to your research, read the appropriate section before selecting a response.

Materials & experimental systems

n/a	Involved in the study
☒	☐ Antibodies
☒	☐ Eukaryotic cell lines
☒	☐ Palaeontology and archaeology
☒	☐ Animals and other organisms
☒	☐ Clinical data
☒	☐ Dual use research of concern

Methods

n/a	Involved in the study
☒	☐ ChIP-seq
☒	☐ Flow cytometry
☒	☐ MRI-based neuroimaging
