# Supplementary figures and images for: Aspergillus fumigatus strains that evolve resistance to the agrochemical fungicide ipflufenoquin in vitro are also resistant to olorofim
Source: Nat Microbiol. 2023 Dec 27;9(1):29–34. doi: 10.1038/s41564-023-01542-4 (PMC10769868; doi:10.1038/s41564-023-01542-4)

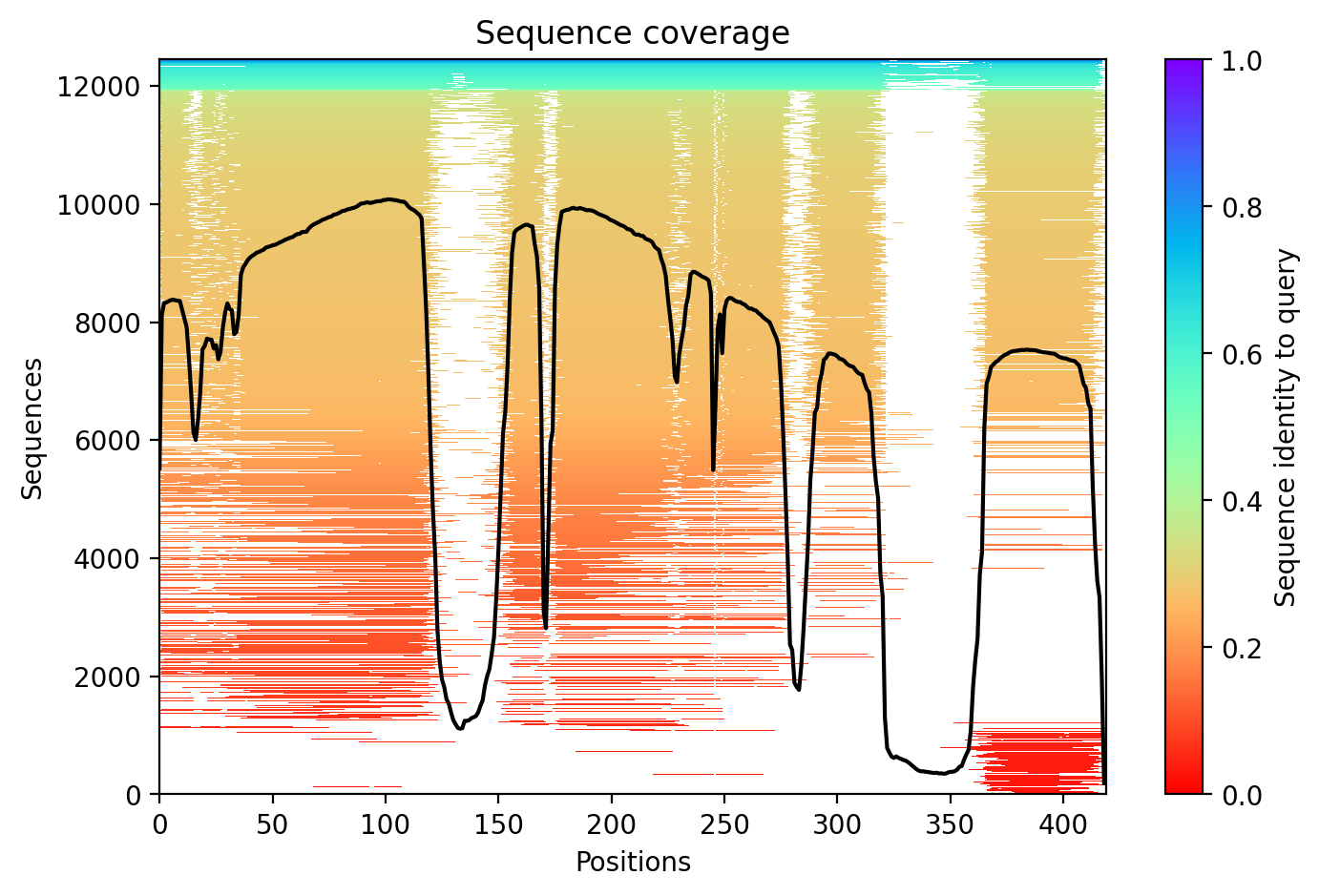

Supplement: Supplementary file 3 — Alphafold2 model of DHODH. [file 41564_2023_1542_MOESM3_ESM.zip › DHODH_D111_L523_wt/DHODH_D111_L523_wt_coverage.png]

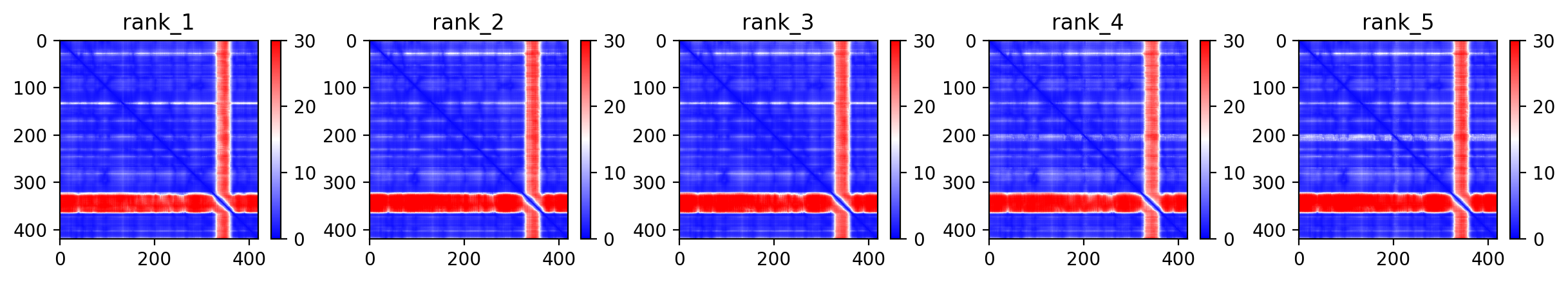

Supplement: Supplementary file 3 — Alphafold2 model of DHODH. [file 41564_2023_1542_MOESM3_ESM.zip › DHODH_D111_L523_wt/DHODH_D111_L523_wt_pae.png]

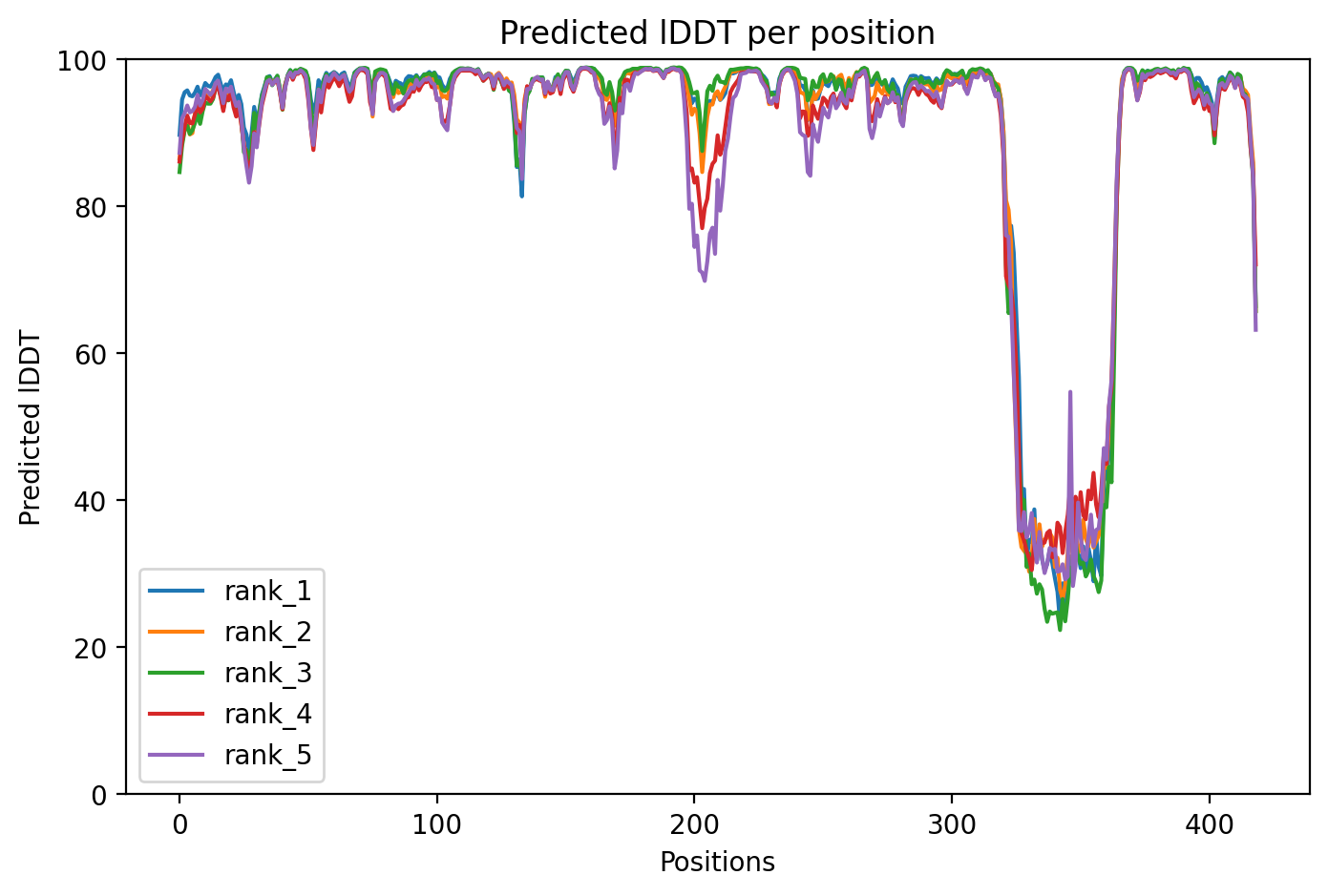

Supplement: Supplementary file 3 — Alphafold2 model of DHODH. [file 41564_2023_1542_MOESM3_ESM.zip › DHODH_D111_L523_wt/DHODH_D111_L523_wt_plddt.png]
